# A Novel Smooth Variable Structure Smoother for Robust Estimation

**DOI:** 10.3390/s20061781

**Published:** 2020-03-23

**Authors:** Yu Chen, Luping Xu, Bo Yan, Cong Li

**Affiliations:** 1 School of Aerospace Science and Technology, Xidian University, Xi’an 710126, China; yu-chen@stu.xidian.edu.cn (Y.C.); boyan@xidian.edu.cn (B.Y.); 2 Department of Electrical, Electronic, and Information Engineering, University of Bologna, 47521 Cesena (FC), Italy; 3 Academy of Space Electronic Information Technology, Xi’an 710100, China; lcongxd@126.com

**Keywords:** robust estimation, smooth variable structure filter, Kalman smoother, target tracking, uncertain system

## Abstract

The smooth variable structure filter (SVSF) is a new-type filter based on the sliding-mode concepts and has good stability and robustness in overcoming the modeling uncertainties and errors. However, SVSF is insufficient to suppress Gaussian noise. A novel smooth variable structure smoother (SVSS) based on SVSF is presented here, which mainly focuses on this drawback and improves the SVSF estimation accuracy of the system. The estimation of the linear Gaussian system state based on SVSS is divided into two steps: Firstly, the SVSF state estimate and covariance are computed during the forward pass in time. Then, the smoothed state estimate is computed during the backward pass by using the innovation of the measured values and covariance estimate matrix. According to the simulation results with respect to the maneuvering target tracking, SVSS has a better performance compared with another smoother based on SVSF and the Kalman smoother in different tracking scenarios. Therefore, the SVSS proposed in this paper could be widely applied in the field of state estimation in dynamic system.

## 1. Introduction

The state and parameter estimation of dynamic systems plays a key role in various application fields. In practice, not only the real-time state estimation of the system is required, but also an estimation result with higher precision through the smoother by using additional measurements made after the time of the estimated state vector is necessary [1,2,3,4]. For example, in a radar system, a smoother is used to obtain higher precision track trajectory for the battlefield situation estimation, and to make more accurate estimates for the aircraft performance. Smoothers are generally divided into fixed-point, fixed-interval, and fixed-lag smoothers [5,6,7], depending on attributes of the application, which type of smoothers can be decided to use. 

In a dynamic system, the real-time sampled data contains various interferences and noises, and it is difficult to obtain the true state of the system. In 1960, in the case of linear Gaussian noise, a recursive solution was obtained using the Kalman filter (KF) [8]. This method was deemed to be the best under a linear, unbiased, and minimum mean square error condition. However, the KF estimates are imprecise and unstable when the system is nonlinear. To address these problems, scholars have proposed some different methods. For example, there is the extended Kalman filter (EKF) [9], the unscented Kalman filter (UKF) [10], and the cubature Kalman filter (CKF) [11,12]. Considering a very small region of nonlinear system as a linear system, EKF was proposed, which is equivalent to the first-order Taylor approximation. Compared with EKF, UKF equals to the second-order Taylor approximation without the Jacobian matrix calculating. In contrast, CKF is based on a third-degree spherical-radial cubature rule that provides a set of cubature points scaling linearly with the state-vector dimension to approximate the state expectation and covariance of a nonlinear system. CKF is equivalent to the fourth-order Taylor approximation and is more stable. In the case of nonlinear and non-Gaussian functions, research about the particle filter is very active, but the huge amount of calculation limits its scope of application [13,14].

One of the main problems of the KF-based filters is that in the case of modeling and parameter uncertainties, the performance of estimation is degraded. For example, state noises and measurement noises have heavy-tailed and/or skewed non-Gaussian distributions when measurements have much clutter [15,16,17]. To deal with this unknown noises, some robust methods have been presented [18,19], when the system with a heavy-tailed or a skewed measurement noises, robust Kalman filters have been proposed by employing the Student’s *t* or skew-*t* distribution to model measurement noises [15,20]. The filter based on a detect-and-reject idea when the measurements are partially disturbed by outliers [21]. For improving robustness and performance of EKF, optimization algorithms [22], adaptive step size control method [23] and online expectation-maximization approach [24] are applied, other theory include optimal [25], minimax estimators [26], etc. Above filters have poor performance in the system with an uncertain model, because mismatching exists between the theoretical signal model of those filters and the variational state model of the system, the theoretical model is entirely consistent variable state model, and is effective in estimation. When the system is a hybrid system that contains finite models, the multi-model methods are proposed to estimate the system state and it are different from single model strategies. The popular multi-model methods include the interactive multi-model method and the variable structure interactive multi-model method [27,28,29,30,31]. So far, the state estimation of dynamic systems is a difficult problem when the modeling and parameter are uncertain, which is worth further study.

The smooth variable structure filter (SVSF) was proposed based on variable structure theory in 2007, and it has advantages in dealing with the problem of uncertain modeling and noise interference [32]. Similar to the KF method, the SVSF is also a predictor-corrector strategy, its gain is based on the discontinuous correction gain, and limits the state estimation around the true state trajectory with a small deviation. Thereby it improves the stability and the robustness of the estimation [32]. The SVSF has been known as a sub-optimal filter [10,12], and different methods have been proposed continually to enrich the theory [33,34,35,36], such as a higher-order version and a revised version [37] of the SVSF, where Gadsden derived the state covariance of the SVSF, and introduced opportunity for its application [38]. Reference [10,12] calculated the optimal smoothing layer with state covariance to determine whether to use SVSF filtering or other filtering methods (e.g., EKF, UKF, CKF), and obtained better filtering results. According to previous research, the SVSF has achieved significant development and is applied in various fields, such as vehicle navigation [39,40], battery management [41,42], fault detection and diagnosis [43,44,45], and artificial intelligence [45,46,47]. However, the ability of SVSF to eliminate noises cannot meet the requirement of estimation in many application conditions such as target tracking, which can be improved by a smoother. There are not many works that previously studied the smoothing problem of the SVSF. The earliest related article [48,49] presented a two-pass smoother based on the SVSF (labeled as SVSTPS), which uses the SVSF gain. As mentioned above, the SVSF gain is considered suboptimal under linear cases [10,12]. Therefore, the performance of SVSF smoothing can be further improved.

This paper is inspired by the Kalman smoother [5,6] and infers the proposed SVSS algorithm in the linear case. Unlike using the SVSF gain, the proposed SVSS method is capable of getting a better state estimation by using the innovation sequence and projection theory [8]. Our former work focus on maneuvering target tracking [50,51,52,53], the SVSS is also applied in maneuvering target tracking. The rest of the paper is structured as follows. Section 2 reviews the SVSF. Section 3 expounds on the smooth theories and the SVSS algorithm. Section 4 exhibits the simulation and analysis. Section 5 concludes the main findings in this paper.

## 2. The SVSF Strategies

Sliding-mode control and estimation is a traditional technique [54] and is widely welcomed as the easy implementation and robustness to uncertainty modeling and errors. Based on Sliding-mode concepts, Habibi first proposed the variable structure filter(VSF) in 2003 [54], which is a new predictor-corrector estimator type, that is applied to linear systems. The SVSF, as an improved form of VSF, can be used for linear or non-linear systems [32].

**Figure 1:**
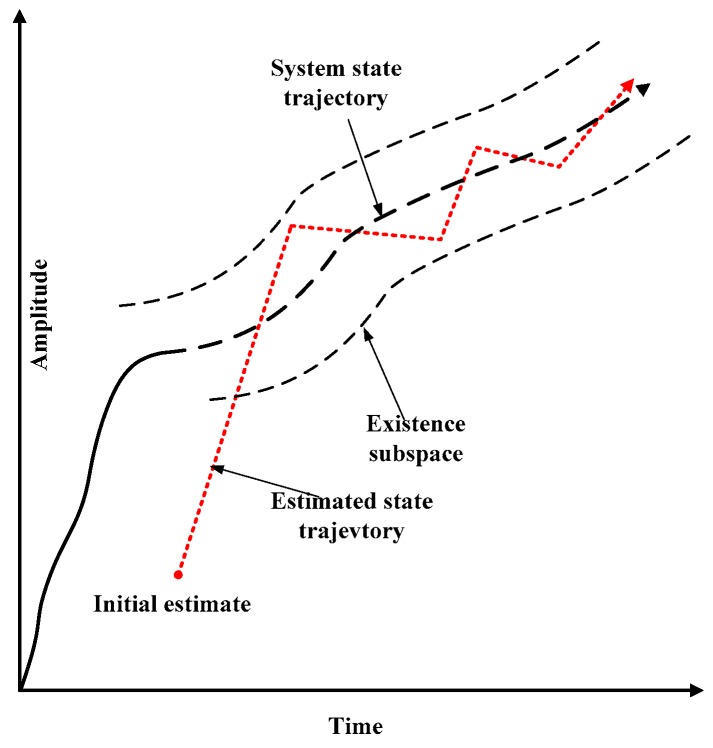
SVSF estimation concept [32].

The trajectory of the state variable over time is shown in Figure 1. In the SVSF concept, the system state trajectory is generated by uncertain modeling. The estimated state trajectory is approximated towards the true trajectory until it reaches the existence subspace. When the estimated state remains in the existence subspace, it is forced to switch back and forth in the true state trajectory. If modeling errors and noises are bounded, then estimates will remain within this limit. The SVSF strategy shows its stability and robustness to modelling uncertainties and errors.

In the case of linear dynamic systems under a zero-mean and Gaussian noise, the state equation and observation equation of a dynamic system are given by Equation (1), respectively:

(1) $$\left\{ \begin{array}{l} x_{k+1}=Fx_{k}+w_{k}\\ z_{k+1}=Hx_{k+1}+v_{k+1} \end{array}\right.$$

The iterative process of SVSF is as follows: 

The predicted state estimation and measurement estimation are calculated as:

(2) $${\hat{x}}_{k+1|k}=F{\hat{x}}_{k|k}$$

(3) $${\hat{z}}_{k+1|k}=H{\hat{x}}_{k+1|k}$$

The measurement innovation is expressed by:

(4) $$e_{k+1|k}=z_{k+1}-{\hat{z}}_{k+1|k}$$

Based on Reference [32], the SVSF gain $K_{k+1}^{SVSF}$ is given by:

(5) $$K_{k+1}^{SVSF}=H^{+}(|e_{k+1|k}|+\gamma |e_{k|k}|)•sat\left({\bar{\psi }}^{-1}e_{k+1|k}\right)$$

where $K_{k+1}^{SVSF}$ is a gain function, $H^{+}$ is the generalized inverse matrix of H,γ (0≤γ<1e) is the SVSF “memory” or convergence rate, |e| is the absolute value of e, and • represents the multiplication of the corresponding elements of the two matrices (Hadamard product). Besides,

(6) $$sat\left({\bar{\psi }}^{-1}e_{k+1|k}\right)=\left\{ \begin{array}{ll} 1, & {\bar{\psi }}^{-1}e_{k+1|k}\geq 1\\ {\bar{\psi }}^{-1}e_{k+1|k}, & -1<{\bar{\psi }}^{-1}e_{k+1|k}<1\\ -1, & {\bar{\psi }}^{-1}e_{k+1|k}\leq -1 \end{array}\right.$$

and

(7) $${\bar{\psi }}^{-1}=\left[\begin{array}{lll} \frac{1}{\psi_{1}} & \;0 & \;0\\ 0\; & \ddots & \;0\\ 0 & 0 & \frac{1}{\psi_{m}} \end{array}\right]$$

where $\psi_{i}$ (i=1,⋯,m) represents the element of a vector ψ that means smoothing boundary value, and m is the measurement dimension. The state estimate is updated by:

(8) $${\hat{x}}_{k+1|k+1}={\hat{x}}_{k+1|k}+K_{k+1}^{SVSF}$$

The posterior measurement estimation and its innovation are described as:

(9) $${\hat{z}}_{k+1|k+1}=H{\hat{x}}_{k+1|k+1}$$

(10) $$e_{k+1|k+1}=z_{k+1}-{\hat{z}}_{k+1|k+1}$$

**Figure 2:**
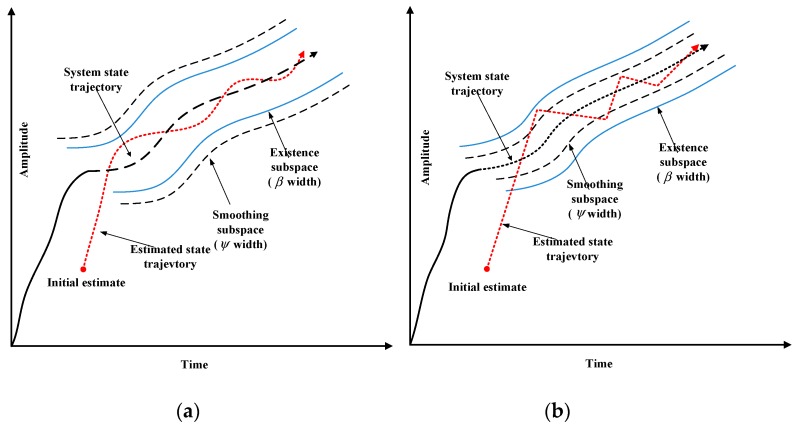
Smoothing boundary layer concept [10,32] (**a**) Smoothed estimated trajectory (ψ≥β); (**b**) Presence of chattering effect (ψ≤β).

The SVSF estimation process converges to the existence subspaces as shown in Figure 1 and Figure 2. The width of the existence subspace β is an uncertain dynamic and time-varying function. In general, the width of β is not completely known, but an upper bound (i.e., ψ) can be chosen based on prior knowledge.

In the existence subspace, the SVSF gain will force the estimated state to switch back and forth along the true state trajectory. The high-frequency switching caused by the gain of the SVSF is called chattering [32], the chattering makes the estimation result inaccurate, and it can be minimized with a given value of smoothing boundary layer ψ. As shown in Figure 2a, when the smoothing boundary layer is larger than the existence subspace boundary layer, the estimated state trajectory is smoothed. When the smoothing boundary layer is smaller than the existence subspace boundary layer, the chattering remains due to the model uncertainty is underestimation, as shown in Figure 2b. Therefore, the SVSF algorithm is an effective estimation strategy for a system that has no an explicit model.

SVSF is generally used to process systems with the same dimension of state estimation and measurement. If the dimension of state estimation is larger than the dimension of measurement, the “artificial” measurement needs to be added [4]. For example, when estimate the speed in target tracking, it is necessary to increase the “artificial” measurements of speed.

## 3. Smoothing Theories and the Proposed Algorithms

**Figure 3:**
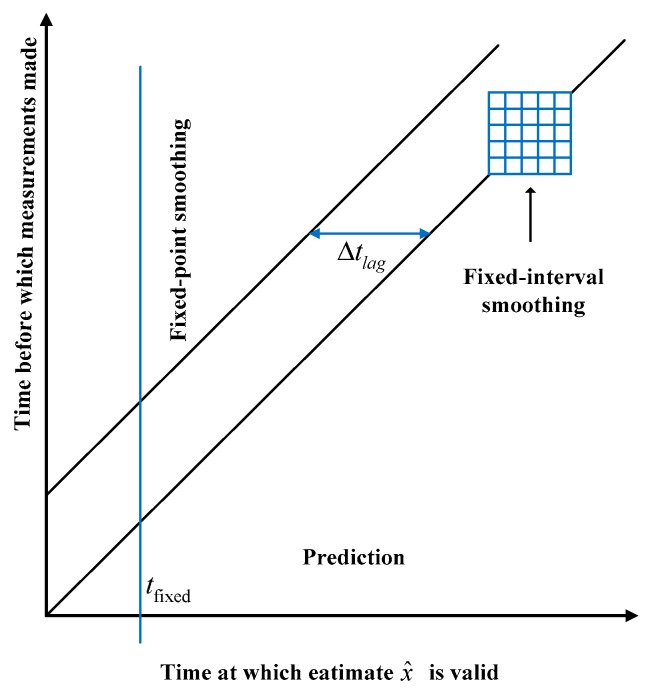
Estimate/measurement timing constraints [55].

Smoothing is a practical strategy using additional measurements made after the time of the estimated state vector to obtain better estimates than those attainable by filtering only. Unfortunately, it is often overlooked. Since the KF algorithm was proposed in 1960 [8], the optimal smoothing method has been derived from the same model using the Kalman theory [55]. Smoothers are the algorithmic or analog implementations of smoothing methods. The relation among smoother, predictor and filter is illustrated in Figure 3, fixed-interval smoothers, fixed-lag smoothers, and fixed-point smoothers are the most typical smoothers, since they have been developed to solve different types of application problems. The fixed-interval smoother is suitable for the trajectory optimization problems. 

SVSF is a novel model-based robust state estimation method and has efficient performance in reducing the disturbances and uncertainties. However, SVSF is less efficient in noise elimination compared to the Kalman filter [10]. Therefore, if its performance in noise canceling can be improved, SVSF would be developed further and be widely utilized in the state estimation field. Previous research indicates that the influence of noises can be eliminated effectively by a filter followed by an additional smoothing process. Therefore, this paper studies the fixed-interval smoother based on SVSF.

### 3.1. The Kalman Smoother 

The popular Rauch–Tung–Striebel (RTS) Two-pass Smoother is one of the fixed-interval Kalman smoothers [55], which is labeled as KS. KS is widely used for its fast iteration and high precision performance, and its iterative process is described below. When the current measurements are obtained, system state and covariance calculated by the Kalman filter in real-time, then, they are used in the smoothing process to obtain a more accurate estimate [55].

(11) $${\hat{x}}_{k+1|k}=F{\hat{x}}_{k|k}$$

(12) $$P_{k+1|k}={FP}_{k|k}F^{T}+Q_{k+1}$$

(13) $${\hat{z}}_{k+1|k}=H{\hat{x}}_{k+1|k}$$

(14) $$S_{k+1}={HP}_{k+1|k}H^{T}+R_{k+1}$$

(15) $$K_{k+1}=P_{k+1|k}H^{T}{}_{k+1}S^{-1}{}_{k+1}$$

(16) $$\left\{ \begin{array}{l} {\hat{x}}_{k+1|k+1}={\hat{x}}_{k+1|k}+K_{k+1}[Z_{k+1}-{\hat{z}}_{k+1|k}]\\ P_{k+1|k+1}=(I-K_{k+1}H)P_{k+1|k}{\left(I-K_{k+1}H\right)}^{T}+K_{k+1}R_{k+1}K_{k+1}^{T} \end{array}\right.$$

The smoothing process is operated backward from the last estimation ${\hat{x}}_{N|N}$ to the first one [55], and the corresponding iterations are given by:

(17) $$\left\{ \begin{array}{l} {\hat{x}}_{k|N}={\hat{x}}_{k|k}+A_{k}({\hat{x}}_{k+1|N}-{\hat{x}}_{k+1|k})\\ A_{k}=P_{k|k}F^{T}P_{k+1|k}^{-1} \end{array}\right.$$

Equations (11)–(17) summarize the Rauch–Tung–Striebel (RTS) Two-pass Smoother (the KS algorithm), which is an iterative process.

### 3.2. The Proposed SVSS Algorithm

The covariance does not necessarily have to be calculated in SVSF, but it is significant for the smoother [38]. The linear system is described by Equation (1). Similar to the calculation method of KF, the predicted state covariance matrix of SVSF is shown as;

(18) $$P_{k+1|k}=FP_{k|k}F^{T}+Q_{k}$$

where $P_{k|k}$ represents the posterior state covariance at k time, and $F^{T}$ represents the system transition matrix, Q is the white noise involved in the system and is calculated by:

(19) $$\left\{ \begin{array}{l} w_{k}\sim N(0,Q_{k})\\ E\left\{w_{k}\right\}=E\left\{w_{k}{}^{T}\right\}=0\\ E\left\{w_{k}w_{k}{}^{T}\right\}=Q_{k}\\ E\left\{w_{k}{\tilde{x}}_{k|k}{}^{T}\right\}=E\left\{w_{k}\right\}E\left\{{\tilde{x}}_{k|k}{}^{T}\right\}=0\\ E\left\{{\tilde{x}}_{k|k}w_{k}{}^{T}\right\}=E\left\{{\tilde{x}}_{k|k}\right\}E\left\{w_{k}^{T}\right\}=0 \end{array}\right.$$

The state estimate ${\hat{x}}_{k+1|k}$ is given by

(20) $${\hat{x}}_{k+1|k}=Fx_{k|k}$$

According to updated estimates ${\hat{x}}_{k+1|k}$ and real measurement $z_{k}$, the innovation can be expressed by:

(21) $$e_{k+1|k}=z_{k}-H{\hat{x}}_{k+1|k}$$

The state estimate is updated as:

(22) $${\hat{x}}_{k+1|k+1}={\hat{x}}_{k+1|k}+K_{k+1}e_{k+1|k}$$

(23) $$K_{k+1}=H^{+}diag\left(\left|e_{k+1|k}\right|+\gamma \left|e_{k|k}\right|\right)•sat\left({\bar{\psi }}^{-1}e_{k+1|k}\right)\times {\left[diag\left(e_{k+1|k}\right)\right]}^{-1}$$

The updated measurement innovation is calculated by:

(24) $$e_{k+1|k+1}=z_{k+1}-H{\hat{x}}_{k+1|k+1}$$

The posterior state covariance $P_{k+1|k+1}$ at k+1 time can be written in:

(25) $$\begin{array}{l} P_{k+1|k+1}=E\left\{{\tilde{x}}_{k+1|k+1}{\tilde{x}}_{k+1|k+1}{}^{T}\right\}\\ =E\left\{\left[\left(I-K_{k+1}H\right){\tilde{x}}_{k+1|k}-K_{k+1}v_{k+1}\right]{\left[\left(I-K_{k+1}H\right){\tilde{x}}_{k+1|k}-K_{k+1}v_{k+1}\right]}^{T}\right\}\\ =E\{[\left(I-K_{k+1}H\right){\tilde{x}}_{k+1/k}{\left({\tilde{x}}_{k+1|k}\right)}^{T}{\left(I-K_{k+1}H\right)}^{T}-\left(I-K_{k+1}H\right){\tilde{x}}_{k+1|k}v_{k+1}^{T}K_{k+1}^{T}\\ \;\;\;\;-K_{k+1}v_{k+1}{\left({\tilde{x}}_{k+1/k}\right)}^{T}{\left(I-K_{k+1}H\right)}^{T}+K_{k+1}v_{k+1}v_{k+1}^{T}K_{k+1}^{T}]\} \end{array}$$

The relationship between the measurement noises and state estimates is depicted as Equation (26) under the linear Gaussian system:

(26) $$\left\{ \begin{array}{l} E\left\{v_{k+1}\right\}=E\left\{v_{k+1}^{T}\right\}=0\\ E\left\{v_{k+1}v_{k+1}^{T}\right\}=R_{k+1}\\ E\left\{{\tilde{x}}_{k+1|k}v_{k}^{T}\right\}=E\left\{{\tilde{x}}_{k+1|k}\right\}E\left\{v_{k}^{T}\right\}=0\\ E\left\{v_{k}{\tilde{x}}_{k+1|k}^{T}\right\}=E\left\{v_{k}\right\}E\left\{{\tilde{x}}_{k+1|k}^{T}\right\}=0 \end{array}\right.$$

where $R_{k+1}$ means the measurement noise covariance matrix. In addition, $v_{k+1}$ follows a Gaussian distribution, i.e.

(27) $$v_{k+1}\sim N\left(v;0,R_{k+1}\right)$$

According to Formulas (25) and (26), the Equation (25) can be rewritten as:

(28) $$P_{k+1|k+1}=\left(I-K_{k+1}H\right)P_{k+1|k}{\left(I-K_{k+1}H\right)}^{T}+K_{k+1}R_{k+1}K_{k+1}^{T}=(I-K_{k+1}H)P_{k+1k}$$

The Equations (18)–(28) are the specific processes of the SVSF algorithm with the iterative update process of the covariance matrix. 

The earliest article about SVSF [48] presented a two-pass smoother (labeled as SVSTPS) based on the Rauch–Tung–Striebel (RTS) Two-pass Smoother. The smoothing process uses Equation (17), in other words, it is based on the SVSF gain directly. However, the estimation result is not satisfying, so the SVSS using innovation sequences is proposed here. The innovation sequence is an orthogonal sequence containing the same statistical information as the original random sequence, so the innovation sequence is a white noise sequence with a zero mean. The smoothing process in the proposed smoother (SVSS) is updated by the innovation sequence according to the projective theorem (minimum mean square error criterion), and has better performance in eliminating Gaussian noise. The recursive equation is related with the smoothed estimates ${\hat{x}}_{k|N}$ and ${\hat{x}}_{k|N-1}$ (with k<N), as follows:

(29) $$\begin{array}{l} {\hat{x}}_{k|N}={\hat{x}}_{k|N-1}+M_{k|N-1}(z_{N}-H{\hat{x}}_{N|N-1})\\ ={\hat{x}}_{k|N-1}+(\prod_{i=k}^{N-1}C_{i})B_{N}(z_{N}-H{\hat{x}}_{N|N-1}) \end{array}$$

where $C_{i}$ and $B_{N}$ are calculated by $C_{i}=P_{i|i}F^{T}P_{i+1|i}^{-1}$ and $B_{N}=P_{N|N-1}H^{T}{\left(HP_{N|N-1}H^{T}+R\right)}^{-1}$ respectively.

**Proof.**  When N=k+n and $e_{k+n}z_{n}-H{\hat{x}}_{N|N-1}$, Equation (29) can be written as:

(30) $${\hat{x}}_{k|k+n}={\hat{x}}_{k|k+n-1}+M_{k|k+n-1}e_{k+n}$$

where the smoothing gain $M_{k|k+n-1}$ is defined in projection theory as:

(31) $$M_{k|k+n-1}=E((x_{k}e_{k+n}^{T})){\left\{E(e_{k+n}e_{k+n}^{T})\right\}}^{-1}$$

And according to Equation (1) and Equation (21), the innovation is given by:

(32) $$e_{k+n}=H(F{\tilde{x}}_{k+n-1|k+n-1}+w_{k+n})+v_{k+n}=H{\tilde{x}}_{k+n|k+n-1}+v_{k+n}$$

Furthermore, according the References [56], the true state error is calculated by:

(33) $${\tilde{x}}_{k+n-1|k+n-1}=F(k+n-1,k){\tilde{x}}_{k|k}+\sum_{i=k+1}^{i+n-1}F(k+n-1,i)\left\{\left[I_{n}-K_{i}H\right]w_{i-1}-K_{i}v_{i}\right\}$$

where F(k+n−1,k+n−1) and F(k+n−1,i) are defined as:

(34) $$F(k+n-1,k+n-1)=I_{n}$$

(35) $$F(k+n-1,i)=\left[I_{n}-K_{k+n-1}H\right]F\cdots \left[I_{n}-K_{i+1}H\right]F$$

Suppose n > 0, then according to Equation (19) and Equation (26), when $w_{k+n-1}⊥x_{k}$ and $v_{k+n}⊥x_{k}$, the expectation can be calculated as:

(36) $$E((x_{k}e_{k+n}^{T}))=E\left[x_{k}{\tilde{x}}_{k+n-1|k+n-1}^{T}\right]F^{T}H^{T}$$

The true state value is expressed as $x_{k}={\hat{x}}_{k|k}+{\tilde{x}}_{k|k}$ and ${\hat{x}}_{k|k}⊥{\tilde{x}}_{k|k}$, substituting (33) into (36) yields

(37) $$\begin{array}{l} E((x_{k}e_{k+n}^{T}))=P_{k|k}F^{T}(k+n-1)F^{T}H^{T}\\ =P_{k|k}\left\{\prod_{i=1}^{n-1}F^{T}{\left[I_{n}-K_{k+i}H\right]}^{T}\right\}F^{T}H^{T}\\ =P_{k|k}F^{T}\left\{\prod_{i=1}^{n-1}{\left[I_{n}-K_{k+i}H\right]}^{T}F^{T}\right\}H^{T} \end{array}$$

From Equation (28), Equation (37) can be written in:

(38) $$E((x_{k}e_{k+n}^{T}))=P_{k|k}F^{T}(\prod_{i=1}^{n-1}P_{k+i+1|k+i}^{-1}P_{k+i+1|k+i+1}F^{T})H^{T}=(\prod_{i=k}^{k+n-1}(P_{i|i}F^{T}P_{i+1|i}^{-1})P_{k+n|k+n-1}H^{T}$$

(39) $$E(e_{k+n}e_{k+n}^{T})={\left(HP_{k+n|k+n-1}H^{T}+R\right)}^{-1}$$

Substituting Equation (38) and Equation (39) into Equation (30) yields:

(40) $$M_{k|k+n-1}=E((x_{k}e_{k+n}^{T})){\left\{E(e_{k+n}e_{k+n}^{T})\right\}}^{-1}=(\prod_{i=k}^{k+n-1}(P_{i|i}F^{T}P_{i+1|i}^{-1})P_{k+n|k+n-1}H^{T}{\left(HP_{k+n|k+n-1}H^{T}+R\right)}^{-1}$$

So Equation (30) is written as:

(41) $${\hat{x}}_{k|k+n}={\hat{x}}_{k|k+n-1}+(\prod_{i=k}^{k+n-1}(P_{i|i}F^{T}P_{i+1|i}^{-1})P_{k+n|k+n-1}H^{T}{\left(HP_{k+n|k+n-1}H^{T}+R\right)}^{-1}e_{k+n}$$

When N=k+n and $e_{k+n}=z_{n}-H{\hat{x}}_{N|N-1}$. Equation (41) can be written as:

(42) $${\hat{x}}_{k|N}={\hat{x}}_{k|N-1}+(\prod_{i=k}^{N-1}C_{i})B_{N}(z_{N}-H{\hat{x}}_{N|N-1})$$

where $C_{i}$ and $B_{N}$ are calculated by $C_{i}=P_{i|i}F^{T}P_{i+1|i}^{-1}$,$B_{N}=P_{N|N-1}H^{T}{\left(HP_{N|N-1}H^{T}+R\right)}^{-1}$. □

The other detailed derivation is described in the Appendix A. Moreover, the validity of this derivation process could be verified from Reference [6].

Equations (18)–(29) summarize the SVSS algorithm proposed in this paper. The pseudo-code is patched as follows.
**The SVSS Algorithm** *Input {*$x_{0},P_{0},\psi ,F,Q,R,H$*} and the sequence measurement {*$z_{1},z_{2},\ldots ,z_{N}$}  ***Step 1 fiter***  ${\hat{x}}_{k+1|k}=Fx_{k|k}$  $P_{k+1|k}=FP_{k|k}F^{T}+Q_{k}$  $e_{k+1|k}=z_{k}-H{\hat{x}}_{k+1|k}$  $K_{k+1}=H^{+}diag\left(\left|e_{k+1|k}\right|+\gamma \left|e_{k|k}\right|\right)•sat\left({\bar{\psi }}^{-1}e_{k+1|k}\right)\times {\left[diag\left(e_{k+1|k}\right)\right]}^{-1}$  ${\hat{x}}_{k+1|k+1}={\hat{x}}_{k+1|k}+K_{k+1}e_{k+1|k}$  $P_{k+1|k+1}=\left(I-K_{k+1}H\right)P_{k|k+1}{\left(I-K_{k+1}H\right)}^{T}+K_{k+1}R_{k+1}K_{k+1}^{T}$  $e_{k+1|k+1}=z_{k+1}-H{\hat{x}}_{k+1|k+1}$
  ***Step 2 smoothing***  $C_{i}=P_{i|i}F^{T}P_{i+1|i}^{-1}$  $B_{N}=P_{N|N-1}H^{T}{\left(HP_{N|N-1}H^{T}+R\right)}^{-1}$  ${\hat{x}}_{k|N}={\hat{x}}_{k|N-1}+(\prod_{i=k}^{N-1}C_{i})B_{N}(z_{N}-H{\hat{x}}_{N-1})$  *Output {*${\hat{x}}_{k|N}$}

## 4. Simulation

### 4.1. A Classic Target Tracking Scenario

**Figure 4:**
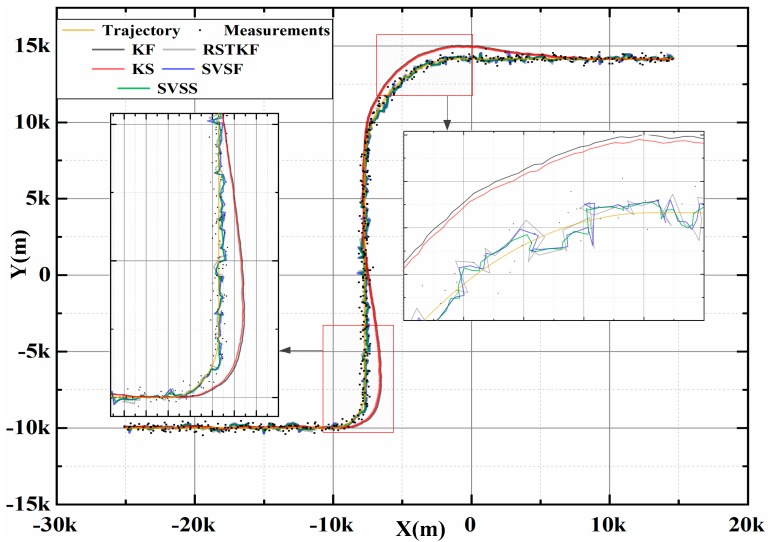
Position trajectory of one experiment.

A classic simulation scenario is always used in References [10,30,57]. This is a tracking problem with a generic air traffic control (ATC) scenario: The target position is provided by a radar system. The system noise obeys a Gaussian distribution with  200 m standard deviation. Figure 4 shows that an aircraft spent 125 s to move from the initial position of  [−25000m,−10000m] at t=0s, and flew with a speed of 120 m/s along the x-axis direction and remained 0m/s along the y-axis direction. Then it turned at a rate of $3^{{}^{\circ}}/s$ for 30s. Next, the target flew in a straight line for 125s, and maneuvered at a rate of $1^{{}^{\circ}}/s$ for 90s. Finally the target flew straight for 120s till the end. 

In the ATC scenario, the behavior of the civilian aircraft could be modeled by a uniform motion (UM) [10,30,57], i.e.,

(43) $$x_{k+1}=\left[\begin{array}{l} 1\;T\;0\;0\\ 0\;1\;0\;0\\ 0\;0\;1\;T\\ 0\;0\;0\;1 \end{array}\right]x_{k}+\left[\begin{array}{l} \frac{1}{2}T^{2}\;0\\ 0\;T\\ \frac{1}{2}T^{2}\;0\\ 0\;T \end{array}\right]w_{k}$$

where T refers to the sampling rate and $w_{k}$ denotes the system noise. The state vector $x_{k}$ of the systems is defined by:

(44) $$x_{k}={\left[\eta_{k},{\dot{\eta }}_{k},\xi_{k},{\dot{\xi }}_{k}\right]}^{T}$$

where $\eta_{k}$ and $\xi_{k}$ represent the position of the aircraft along the x-axis and y-axis directions, ${\dot{\eta }}_{k}$ and ${\dot{\xi }}_{k}$ represent its speed, respectively. In the target tracking processing system, generally, radar only provides position measurements without target speed measurements. Therefore, the measurement model can be expressed as:

(45) $$z_{k}=\left[\begin{array}{l} 1\;0\;0\;0\\ 0\;0\;1\;0 \end{array}\right]x_{k}+v_{k}$$

In the simulation, the state of the filter parameter is initialized as $x_{0}=[-25000,80,-10000,-10]$. Moreover, measurement covariance R, state covariance $P_{0|0}$ and process noise covariance Q are set as follows:

(46) $$R=200^{2}\left[\begin{array}{l} 1\;0\\ 0\;1 \end{array}\right]$$

(47) $$P_{0|0}diag([10000,1000,10000,1000])$$

(48) $$Q=L*\left[\begin{array}{llll} \frac{\;T^{3}}{3} & \frac{T^{2}}{2} & \;0 & \;0\\ \frac{T^{2}}{2} & T & \;0 & \;0\\ \;0 & \;0 & \frac{\;T^{3}}{3} & \frac{T^{2}}{2}\\ \;0 & \;0 & \frac{T^{2}}{2} & T \end{array}\right]$$

where L represents the power spectral densities and is defined as 0.16 [10,30,57]. Besides, the SVSF “memory” ( i.e. convergence rate) was set to γ=0.1, which is tuned based on some knowledge of the system uncertainties such as the noise to decrease the estimation errors [58]. Those methods are coded with MATLAB and the simulations are run on a laptop computer with Intel Core i5-3230M CPU at 2.40 GHz. Five hundred trials of Monte Carlo are performed in each simulation, and the estimation results are expressed in the figures of merit-root mean square error (RMSE), accumulative RMSE and the average value.

1 Compared with KF

In this simulation, Kalman filter (KF), Kalman smoother (KS), Robust Student’s t-Based Kalman Filter (RSTKF) [20], SVSF and the proposed SVSF are tested. The SVSF and SVSS of smooth boundary layer widths is set to ψ=[800,800] m, the In the RSTKF, the prior parameters are set as: w=v=5,τ=5,N=10[20].

**Figure 5:**
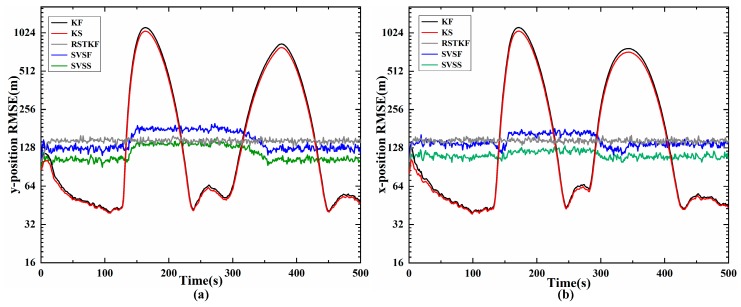
Position of RMSE of x-axis and y-axis (m) (**a**) position of RMSE on x-axis; (**b**) position of RMSE on y-axis.

Figure 4 shows the target tracking position trajectory of one experiment, so the closer the tracking trajectory is to the real trajectory, the more accurate the tracking method is. The trajectory of SVSS is closer to true trajectory than SVSF, so SVSS reduces the chattering phenomena. The state estimation results of KF, KS, SVSF and the proposed SVSS are shown in Figure 5 and Table 1. Compared with RSTKF, SVSF and SVSS, when the system modeling is in accordance with target motions, the KF and KS exhibit better performance. But when the target motion model changes, a dramatic increase occurs in the tracking errors of KF and KS, while the estimations of RSTKF, SVSF and SVSS still maintain high accuracy. In Figure 5, the aircraft flies in a straight line after 155 s, the RMSE of SVSF does not decrease because the SVSF algorithm could not directly estimate the speed information. Therefore, there are no modifications in the relevant speed information. How to effectively use the “artificial” velocity measurements to modify the speed information will be described in the following. As shown in Figure 5 and Table 1, the simulation results demonstrate that for both KF and SVSF, the tracking errors obtained from their smoothers are smaller than that from the filters. In addition, Table 1 shows the position accumulative RMSE of SVSS is the lowest among KF, KS, RSTKF and SVSF. SVSS improves tracking accuracy by about 22% compared with SVSF and RSTKF, while KS only improves about 7% compared with KF. The reason for phenomenon is that the ability of SVSF to deal with noise is weaker than KF, so SVSS is easier to eliminate noises in its state estimates value compared to KS. KS and SVSS in smoothing process consume similar time, SVSF spends a little more time compared to KF, The RSKF cost most time. To sum up, SVSS has better robustness and higher tracking accuracy than the others.

2 Results under different smooth boundary layer widths

**Figure 6:**
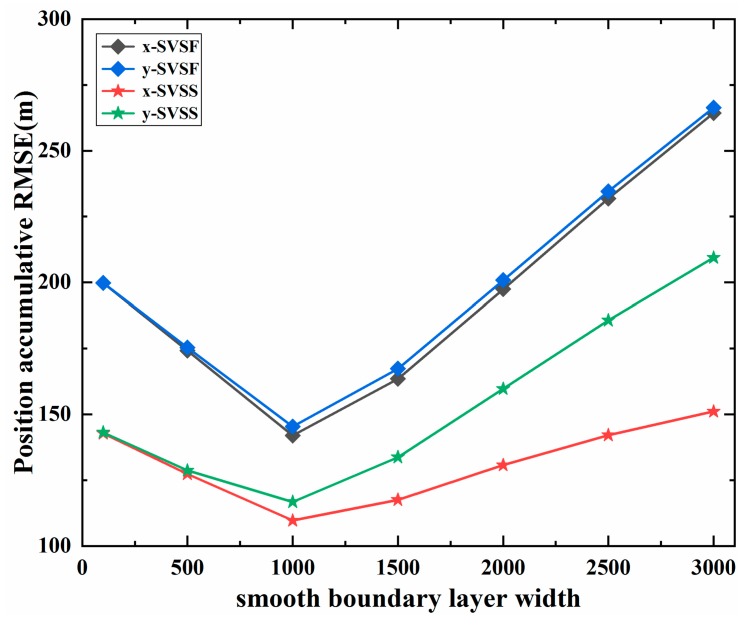
The position accumulative of different ψ on the x-axis and y-axis (m).

Figure 6 and Table 2 show results of the position accumulative RMSE under different smooth boundary layer widths. We can see that SVSS has a better performance than SVSF, because the ability of SVSF to eliminate noise is almost the same under different smooth boundary layer widths. In addition, the smooth boundary layer width ψ is an important parameter in SVSF. If it is not set properly, both the filter accuracy and stability would be deteriorated. The accumulative RMSE of SVSF estimation is high when the smooth boundary layer width is too large or too small. As shown in Figure 6, when the smoothing boundary layer ψ is selected as 1000m, the proposed method has a better performance under the system measurement noises with standard deviation of 200m. These parameters are selected based on the distribution of the system and measurement noises. Generally, the smoothing boundary layer width ψ is set to be 5 times the maximum measurement noise width. And the SVSF “memory” or convergence rate γ(0≤γ<1) is related to the rate of decay α, such that α=(1/τ)ln(γ),where the τ is the sampling time [32]. As shown in Figure 6, when the width of smooth boundary layer is large, the accumulated RMSE of SVSS position on x-axis and y-axis is different, the reason is the same as above, there is no modifications in the relevant speed.

3 Results under different lag fixed intervals

Simulations under different lag fixed intervals are also considered. The width of the smooth boundary layer is ψ=[800,800] m, and the other experimental parameters are the same as mentioned above.

**Figure 7:**
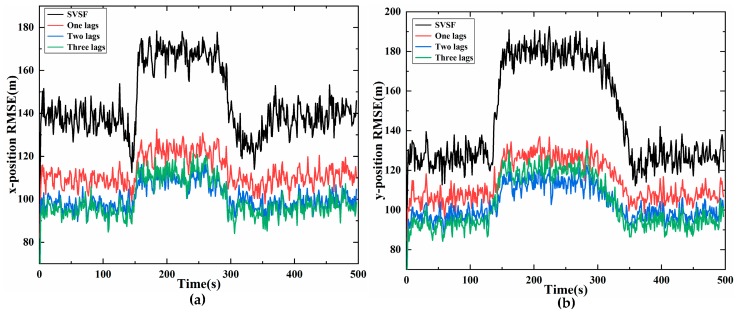
SVSF smoother of different lags compare (**a**) position RMSE on x- axis; (**b**) position RMSE on y-axis.

Figure 7 shows the estimation results using SVSS under three different lags and SVSF, and the performance of two lags and three lags is better than that of one lag. Compared with two lags, when the target moves with a constant velocity, the three lags improves the tracking accuracy slightly, but when the target makes a turning movement, the accuracy of three lags is lower than that of two lags. As a matter of fact, when the system model is consistent with the actual model, the proposed SVSS, which is derived under linear Gaussian noise, this can increase the estimation accuracy as does increasing the smoothing lag steps. However, if the models are inconsistent, the performance of the SVSS will probably decrease and is unstable as lag steps of smoothing increase, because innovation information in the SVSS contains more modeling error innovation. With the increase of the lag of SVSS, the computational complexity becomes higher. The computational complexity of n lags is $O\left(n\left(n-1\right)/2*\left(3+m\right)i^{3}+2nji^{2}+2nij^{2}+nmj^{3}\right)$, and the detailed derivation is described in the Appendix B, the simulation time cost of different lags is also shown in Table A2 of the Appendix B. Therefore, under different requirements, users can choose the appropriate lag steps according to the needs of the system.

### 4.2. A Complex Maneuvering Environment Scenario

As for this scenario, the maneuver of the target is more complicated, motion modes including uniform motion, turning motion, uniform acceleration motion and angular acceleration motion. The initial position value of the aircraft is also set to be  [−25000m,−10000m]. Target flies with a high speed of 300m/s along the x-axis direction and 250m/s along the y-axis direction for 20s.Then the aircraft maneuvers at a rate of $-3^{{}^{\circ}}/s$ for 55s. Next, the target turns at an initial angular velocity of $1^{\circ }/s$ with an angular acceleration of $0.5^{\circ }/s^{2}$ for 25s. Finally the target continues to fly at an acceleration of $20m/s^{2}$ along the x-axis direction and $10m/s^{2}$ along the y-axis direction for 25s. 

The process noises obeys a Gaussian distribution with 100m standard deviation. The process noises and measurement noises (w and v) are considered as Gaussian, with a zero mean and variances Q and R, respectively. The initial state covariance $P_{0|0}$, measurement noise covariance R, and process noise covariance Q are defined, respectively, as follows:

(49) $$R=100^{2}\left[\begin{array}{l} 1\;0\\ 0\;1 \end{array}\right]$$

(50) $$P_{0|0}=diag([400,100,400,100])$$

where Q is given by the Equation (48) and L is defined as 15. For the SVSF and their smoother estimation processes use the UM model, and smoothing boundary layer widths were defined as, $\psi ={\left[500,600,500,600\right]}^{T}$; the SVSF “memory” or convergence rate was set to γ=0.1. In order to meet the actual demand, the estimation of speed is increased. As per earlier discussions, the system also needs to add an “artificial” velocity measurement; it is necessary to transform the measurement matrix of (45) into a square matrix (i.e., identity), and the “artificial” velocity measurement can be calculated by position measurements. For example, where $y_{k}$ represents measurement, which contains the artificial velocity measurements:

(51) $$y_{k}={\left[z_{1,k},(z_{1,k}-z_{1,k-1})/T,z_{2,k},(z_{2,k}-z_{2,k-1})/T\right]}^{T}$$

The accuracy of Equation (51) depends on the sampling rate T. A total of 500 Monte Carlo runs were performed.

**Figure 8:**
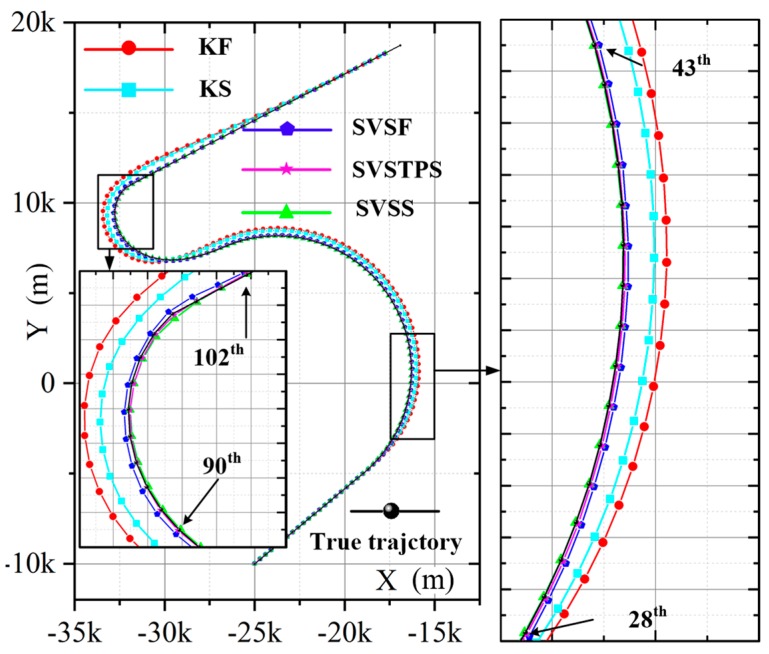
Average position trajectory.

**Figure 9:**
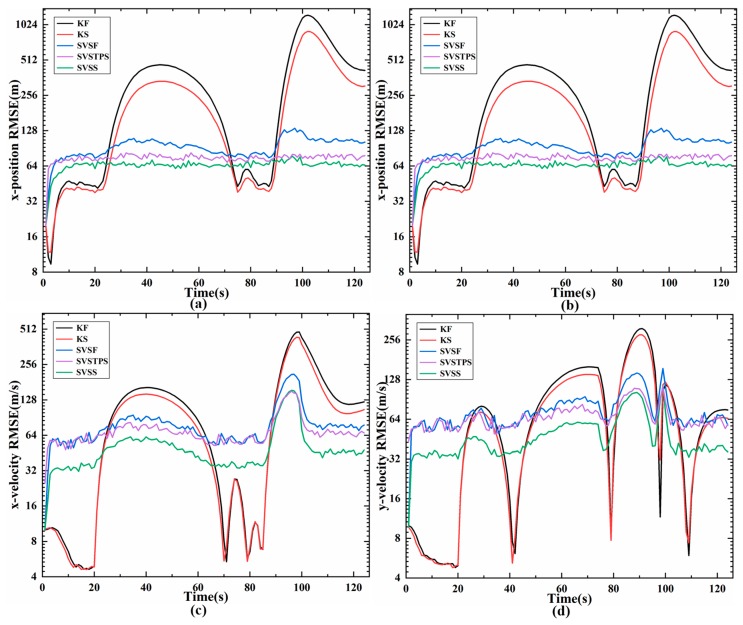
RMSE of state estimation (m) (**a**) position RMSE on x- axis; (**b**) position RMSE on y-axis; (**c**) velocity RMSE on x- axis; (**d**) velocity RMSE on y-axis.

The results of different estimation methods are shown in the following figures and Table 3, so we can see that the KS algorithm has the best performance when the model is matched (0 s to 20 s) with the actual motions, and the position RMSE and speed RMSE of KS are significantly smaller than the other two SVSF smoothers. Once the models do not match, such as the high speed target change from uniform motion to turn, angular acceleration, or acceleration, KS is unstable and RMSE increases, but the SVSTPS and SVSS are still able to overcome the uncertainties and have high accuracy and robustness. Table 3 shows that the position (velocity) accumulative RMSE of SVSTPS and SVSS increased by about 21% (15%) and 31% (33%) than SVSF, respectively. This is because SVSTPS uses SVSF gain, and the proposed SVSS uses innovation, and innovation is more conducive to smoother processing due to it containing as much noises as the original measurements. Thus more noise errors can be eliminated by SVSS. However, there is a special case where the SVSS performs not as well as SVSTPS when modeling errors affect it more than Gaussian noise. For example, at around 95 s in Figure 8 and Figure 9, the target is flying at a high speed with the angular velocity of $12^{\circ }/s$ and angular acceleration of $0.5^{\circ }/s^{2}$, so accuracy of SVSS is lower than that of SVSTPS. Therefore, SVSS exhibits the best performance with the above filters.

## 5. Conclusions 

An SVSS algorithm is presented to improve the accuracy of the SVSF state estimation in the dynamic system with model uncertainty. Based on projection theory and SVSF, the smoothing recurrence formula of SVSS is deduced using innovation. The comparisons among SVSS, KS and SVSTPS are analyzed in the scene of aircraft trajectory tracking. According to the simulation results, the proposed SVSS performs well than SVSF under different bounded smoothing layers. In addition, compared with the popular KS algorithm, the SVSS is able to overcome inaccuracies and yield a stable solution in the presence of modeling uncertainties. Theory and simulations also prove that, the proposed SVSS has higher accuracy and better robustness compared with SVSTPS. 

## Figures and Tables

**Table 1:** The position accumulative RMSE on the x-axis and y-axis (m).

Different Methods	KF	KS	RSTKF	SVSF	SVSS
Position of accumulative RMSE on x-axis (m)	413	387	145	146	113
Position of accumulative RMSE om y-axis (m)	413	387	144	148	117
Single step run time (*μs*)	44	68	1192	48	72

**Table 2:** The position accumulative RMSE on the x-axis and y-axis (m) for different smooth boundary layer widths.

Different Smooth Boundary Layer(m)	100	500	1000	1500	2000	2500	3000
SVSF position accumulative RMSE on x-axis(m)	200	174	142	164	197	232	264
SVSS position accumulative RMSE on x-axis(m)	143	127	110	118	131	142	151
SVSF position accumulative RMSE on y-axis(m)	200	175	145	167	201	235	266
SVSS position accumulative RMSE on y-axis(m)	143	129	117	134	160	186	209

**Table 3:** The state estimation accumulative RMSE on x-axis and y-axis (m) for different algorithms.

State Estimation	KF	KS	SVSF	SVSTPS	SVSS
x-position accumulative RMSE(m)	461	335	95	75	65
x-velocity accumulative RMSE(m/s)	160	141	86	73	57
y-position accumulative RMSE(m)	297	218	92	75	65
y-velocity accumulative RMSE(m/s)	113	102	78	69	52

## Appendix A

Proof process is based on the orthogonal projection theory:

### Appendix A.1. Definition 1

Based on random measurement variables $z\in R^{m}$, estimate variables $\hat{x}\in R^{n}$, which represent the linear minimum variance estimation of $x\in R^{n}$, is a linear function of z, the minimization index of estimate J which is given by the following

(A1) $$J=E[{\left(x-\hat{x}\right)}^{T}(x-\hat{x})]$$

where E is the mean sign, and T is the transpose symbol,

### Appendix A.2. Definition 2

The linear minimum variance estimation is only given by the following formula

(A2) $$\hat{x}=Ex+P_{xz}P_{zz}^{-1}(z-E(z))$$

(A3) $$P_{xz}=E[(x-E(x)){\left(z-E(z)\right)}^{T}]$$

(A4) $$P_{zz}=E[(z-E(z)){\left(z-E(z)\right)}^{T}]$$

*Property 1*

(A5) $$E\hat{x}=Ex$$

*Property 2*

(A6) $$E[(x-\hat{x})z^{T}]=0$$

*Property 3*

Where $x-\hat{x}$ and z are uncorrelated random variables, recorded as $x-\hat{x}⊥z$, called $\hat{x}$ which the projection of x on z.

(A7) $$\hat{x}=proj(x|z)$$

### Appendix A.3. Definition 3

Based on random variables $z_{1},z_{2},\cdots ,z_{k}\in R^{m}$, The linear minimum variance estimate $\hat{x}$ of the random variables $x\in R^{n}$ is defined as:

(A8) $$\hat{x}=proj(x|w)≜proj(x|z_{1},z_{2},\cdots z_{k})$$

estimate $\hat{x}$ is called x projective on the L(w) or $L(z_{1},z_{2},\cdots z_{k})$.

### Appendix A.4. Definition 4

If random variables $z_{1},z_{2},\cdots ,z_{k}\in R^{m}$ has second moment, its innovation is defined as:

(A9) $$e_{k}=z_{k}-proj(z_{k}|z_{1},z_{2},\cdots z_{k-1}),\;k=1,2,\cdots$$

The predicted estimate of ${\hat{z}}_{k|k-1}$ is defined as:

(A10) $${\hat{z}}_{k|k-1}=proj(z_{k}|z_{1},z_{2},\cdots z_{k-1})$$

(A11) $$e_{k}=z_{k}-{\hat{z}}_{k|k-1},\;k=1,2,\cdots$$

(A12) $$e_{k}=z_{k}-H{\hat{x}}_{k|k-1}=H(F{\hat{x}}_{k|k}+w)+v$$

(A13) $$proj(x|z_{1},z_{2},\cdots z_{k})=proj(x|e_{1},e_{1},\cdots e_{k})$$

### Appendix A.5. Definition 5

For the estimate of random variable $\hat{x}$, when the random variable $z_{1},z_{2},\cdots ,z_{k}\in R^{m}$ has the second moment, we have the following iterative projective Formula (A14) (Projective recurrence theorem)

(A14) $$prog(x|z_{1},z_{1},\cdots z_{k})=prog(x|z_{1},z_{1},\cdots ,z_{k-1}+E[(xe_{k}^{T})]{\left(E[(e_{k}e_{k}^{T}))\right)}^{-1}e_{k}$$

The derivation of the SVSS algorithm is also based on the above theory

(1) One lag fixed interval

For the linear Gaussian system given by Equation (1), under the linear minimum mean square deviation criterion, the state estimate value of SVSS is given by the following formula:

(A15) $${\hat{x}}_{k|k+1}={\hat{x}}_{k|k}+M_{k|k+1}e_{k+1}$$

(A16) $$M_{k|k+1}=P_{k|k}F^{T}H^{T}{\left(HP_{k+1|k}H^{T}+R\right)}^{-1}$$

From (A2) to (A15), the state estimate value is obtained as:

(A17) $${\hat{x}}_{k|k+1}={\hat{x}}_{k|k}+P_{xz}(k+1)P_{zz}(k+1)e_{k+1}$$

(A18) $$P_{xz}(k+1)=E[(x_{k}e_{k+1}^{T})]$$

(A19) $$P_{xz}(k+1)=E[(x_{k}e_{k+1}^{T})]=E(x_{k}{\left(H(F{\tilde{x}}_{k|k}+w)+v\right)}^{T})=P_{k|k}F^{T}H^{T}$$

(A20) $$P_{zz}(k+1)=E[(e_{k+1}e_{k+1}^{T})]=HP_{k+1|k}H+R$$

(A21) $${\hat{x}}_{k|k+1}={\hat{x}}_{k|k}+P_{k|k}F^{T}H^{T}{\left(HP_{k+1|k}H^{T}+R\right)}^{-1}e_{k+1}$$

(2) Two lags fixed interval

As one-lag, the state estimate value of SVSS is given by the following formula:

(A22) $${\hat{x}}_{k|k+2}={\hat{x}}_{k|k+1}+M_{k|k+2}e_{k+2}$$

(A23) $${\hat{x}}_{k|k+2}={\hat{x}}_{k|k+1}+P_{xz}(k+2)P_{zz}(k+2)e_{k+2}$$

(A24) $$P_{xz}(k+2)=E[(x_{k}e_{k+2}^{T})]=E(x_{k}{\left(H(F{\tilde{x}}_{k+1|k+1}+w)+v\right)}^{T})=P_{k|k}F^{T}P_{k+1|k}^{-1}P_{k+1|k+1}F^{T}H^{T}$$

(A25) $$P_{zz}(k+2)=E[(e_{k+2}e_{k+2}^{T})]=HP_{k+2|k+1}H+R$$

(A26) $$M_{k|k+2}=P_{k|k}F^{T}P_{k+1|k}^{-1}P_{k+1|k+1}F^{T}H^{T}{\left(HP_{k+2|k+1}H^{T}+R\right)}^{-1}$$

According to (A23)–(A26), the state estimate value of SVSS is calculated by:

(A27) $${\hat{x}}_{k|k+2}={\hat{x}}_{k|k+1}+P_{k|k}F^{T}P_{k+1|k}^{-1}P_{k+1|k+1}F^{T}H^{T}{\left(HP_{k+2|k+1}H^{T}+R\right)}^{-1}e_{k+2}$$

(3) N lags fixed interval

(A28) $${\hat{x}}_{k|k+n}={\hat{x}}_{k|k+n-1}+M_{k|k+n-1}e_{k+n}$$

(A29) $${\hat{x}}_{k|k+n}={\hat{x}}_{k|k+n-1}+P_{xz}(k+N)P_{zz}(k+N)e_{k+n}$$

(A30) $$e_{k+n}=H(F{\tilde{x}}_{k+n-1|k+n-1}+w)+v$$

(A31) $$P_{xz}(k+n)=E[(x_{k}e_{k+n}^{T})]=E(x_{k}{\left(H(F{\tilde{x}}_{k+n-1|k+n-1}+w)+v\right)}^{T})$$

where $P_{xz}(k+n)$ is obtained from the orthogonality of the projection theory:

(A32) $$\begin{array}{l} P_{xz}(k+n)=P_{k|k}(\prod_{i=1}^{n-1}F^{T}P_{k+i+1|k+i}^{-1}P_{k+i+1|k+i+1})F^{T}H^{T}=P_{k|k}F^{T}(\prod_{i=1}^{n-1}P_{k+i+1|k+i}^{-1}P_{k+i+1|k+i+1}F^{T})H^{T}\\ =(\prod_{i=k}^{k+n-1}(P_{i|i}F^{T}P_{i+1|i}^{-1})P_{k+n|k+n-1}H^{T} \end{array}$$

(A33) $$M_{k|k+n}=(\prod_{i=k}^{k+n-1}(P_{i|i}F^{T}P_{i+1|i}^{-1})P_{k+n|k+n-1}H^{T}{\left(HP_{k+n|k+n-1}H^{T}+R\right)}^{-1}$$

Form (A29) to (A33), the n lag state estimate value of SVSS is calculated by:

(A34) $${\hat{x}}_{k|k+n}={\hat{x}}_{k|k+n-1}+(\prod_{i=k}^{k+n-1}(P_{i|i}F^{T}P_{i+1|i}^{-1})P_{k+n|k+n-1}H^{T}{\left(HP_{k+n|k+n-1}H^{T}+R\right)}^{-1}e_{k+n}$$

Equation (A34) can be written as:

(A35) $${\hat{x}}_{k|N}={\hat{x}}_{k|N-1}+(\prod_{i=k}^{N-1}C_{i})B_{N}(z_{N}-H{\hat{x}}_{N|N-1})$$

where $C_{i}$ and $B_{N}$ are calculated by $C_{i}=P_{i|i}F^{T}P_{i+1|i}^{-1}$,$B_{N}=P_{N|N-1}H^{T}{\left(HP_{N|N-1}H^{T}+R\right)}^{-1}$.

## Appendix B

The smoothing process is expressed by Equation (42). The state and the measurement dimension are *i* and *j* respectively, and the complexity of matrix computation is shown in Table A1.

**Table A1:** The complexity of matrix computation.

	Complexity		Complexity
*PF*	*O*(*i*^3^)	*C_i_*	*O*(2*i*^3^ + *mi*^3^)
*PH*	*O*(*ji*^2^)	*B_N_*	*O*(2*ji*^2^ + 2*ij*^2 +^*mj*^3^)
*P* ^−1^	*O*(*mi*^3^)		

Ignoring the low computational complexity, the computational complexity of one lag smoothing is $O\left(i^{3}+2ji^{2}+mj^{3}\right)$.

The computational complexity of n lag smoothing is $O\left(n\left(n-1\right)/2*\left(3+m\right)i^{3}+2nji^{2}+2nij^{2}+nmj^{3}\right)$. The implementation times of SVSS under different lag fixed intervals is shown in Table A2.

**Table A2:** Implementation Times of SVSS under different lag fixed intervals.

Different Lags	SVSF	One Lags	Two Lags	Three Lags
Single step run time (*μs*)	48	72	120	180
